# The Prevalence of Intestinal Parasites and Their Associated Factors among Diabetes Mellitus Patients at the University of Gondar Referral Hospital, Northwest Ethiopia

**DOI:** 10.1155/2020/8855965

**Published:** 2020-11-20

**Authors:** Sintayehu Ambachew, Muluneh Assefa, Yalewayker Tegegne, Ayalew Jejaw Zeleke

**Affiliations:** ^1^Department of Clinical Chemistry, School of Biomedical and Laboratory Sciences, College of Medicine and Health Sciences, University of Gondar, Gondar, Ethiopia; ^2^Department of Medical Microbiology, School of Biomedical and Laboratory Sciences, College of Medicine and Health Sciences, University of Gondar, Gondar, Ethiopia; ^3^Department of Medical Parasitology, School of Biomedical and Laboratory Sciences, College of Medicine and Health Sciences, University of Gondar, Gondar, Ethiopia

## Abstract

**Background:**

Worldwide, more than one-sixth of the population is infected by intestinal parasites, of which the majority live in developing countries. On the other hand, the prevalence of diabetes mellitus has been increasing over recent decades in developing countries. Patients with diabetes mellitus encountered impaired immunity and suffer from the consequences of infection particularly intestinal parasitic infection.

**Objective:**

This study is aimed at assessing the prevalence of intestinal parasites and associated factors among diabetes mellitus patients at the University of Gondar Referral Hospital, Northwest Ethiopia.

**Methods and Materials:**

An institutional-based cross-sectional study was conducted at the University of Gondar Comprehensive Specialized Referral Hospital from February 15 to March 30, 2018. A total of 234 diabetes mellitus patients were enrolled. A systematic random sampling technique was used to select study participants. Sociodemographic and clinical data were collected using a semistructured questionnaire. A 5-gram stool sample was collected to identify parasitic infection using a direct wet mount and formal-ether concentration technique. Data was entered and analyzed by using SPSS version 20. A *p* value of ≤0.05 was considered as statistically significant.

**Result:**

In the current study, the overall prevalence of intestinal parasite infection among diabetics was 45 (19.2%). The parasites identified in this study were *Ascaris lumbricoides* 15 (6.41%), *Entamoeba histolytica/dispar* 9 (3.85%), Hookworm 9 (3.85%), *Schistosoma mansoni* 7 (3%), *Enterobius vermicularis* 3 (1.3%), and *Giardia lamblia* 2 (0.9%). Poor educational background (AOR = 3.62; 95% CI (1.038, 12.65); *p* = 0.043), poor hygiene and sanitation (AOR = 4.67; 95% CI (1.82, 12.07); *p* = 0.001), and inappropriate latrine usage (AOR = 5.41; 95% CI (1.43, 20.56); *p* = 0.013) were significantly associated with the prevalence of intestinal parasitic infection among diabetes mellitus patients.

**Conclusion:**

The overall prevalence of intestinal parasitic infection among diabetes mellitus patients was relatively high. There should be continued prevention, control, and management of intestinal parasitic infection in such a study population.

## 1. Introduction

Intestinal parasites are organisms that live in the gastrointestinal tract of animals, including humans. These are usually transmitted when someone comes in contact with infected feaces [1]. Intestinal worms produce a wide range of symptoms including intestinal manifestations (diarrhea and abdominal pain), general malaise, and weakness. Parasites such as Hookworms cause chronic intestinal blood loss that results in anemia [2]. Factors predisposing to intestinal parasitic infections are poor sanitary conditions such as personal hygiene and environmental sanitation, level of education and occupation, the type and duration of diabetes, inappropriate utilization of latrine, and lack of awareness resulting in the contamination of food and water sources with a consequent continuance of parasite cycles [3]. Intestinal parasitic infections are a major concern, mostly in developing countries, particularly in sub-Saharan Africa. Ethiopia has one of the lowest quality drinking water supply and latrine coverage in the world, and because of this and other risk factors, intestinal parasitic infections are the second most predominant causes of outpatient morbidity in the country [4].

The WHO estimates that about 3.5 billion people are infected and approximately 450 million people are ill as a result of intestinal parasitic infections, the majority being children. In Ethiopia, this infection is among the most common causes of morbidity [5].

Diabetes mellitus is a group of metabolic diseases characterized by hyperglycemia resulting from defects in insulin secretion, insulin action, or both. There are two principal forms of diabetes: Type 1 diabetes (formerly known as insulin-dependent) in which the pancreas fails to produce insulin which is essential for survival. This form develops most frequently in children and adolescents but is being increasingly noted later in life. Type 2 diabetes (formerly named non-insulin-dependent) results from the body's inability to respond properly to the action of insulin produced by the pancreas. Type 2 diabetes is much more common and accounts for around 90% of all diabetes cases worldwide. It occurs most frequently in adults but is being noted increasingly in adolescents as well [6, 7].

Diabetes produces the classical symptoms of polyuria, polydipsia, and polyphagia. The chronic hyperglycemia of diabetes is associated with long-term damage, dysfunction, and failure of different organs, especially the eyes, kidneys, nerves, heart, and blood vessels [6]. The disease burden related to diabetes is high and rising in every country, fuelled by the global rise in the prevalence of obesity and unhealthy lifestyles. The latest estimates show a global prevalence of 382 million people with diabetes in 2013, expected to rise to 592 million by 2035 [8].

Patients with diabetes mellitus have infections more often than those without diabetes mellitus. The course of the infections is also more complicated in this patient group. One of the possible causes of this increased prevalence of infections is defects in immunity. Besides, some decreased cellular responses in vitro, no disturbances in adaptive immunity in diabetic patients have been described. Different disturbances (low complement factor 4 and decreased cytokine response after stimulation) in humoral innate immunity have been described in diabetic patients. Concerning cellular innate immunity, most studies show decreased functions (chemotaxis, phagocytosis, and killing) of diabetic polymorphonuclear cells and diabetic monocytes/macrophages compared to cells of controls [9]. Diabetic patients are considered as the immunocompromised group of patients.

So, this study is aimed at assessing the prevalence of intestinal parasitic infections and associated factors among diabetes mellitus patients. This study helps to be a source of information about the burden on intestinal parasitic infection for those physicians who are working in the diabetes mellitus clinic for early detection, treatment, and prevention of intestinal parasite infection. Moreover, there is a scarcity of data that showed the prevalence of intestinal parasites and its associated factors among diabetes mellitus in Ethiopia. Thus, this study is aimed at providing baseline information for policymakers, researchers, and stakeholders to intervene and provide an appropriate strategy for the control, prevention, and management of intestinal parasitic infection.

## 2. Methods and Materials

### 2.1. Study Area, Design, and Period

A cross-sectional study was conducted at the University of Gondar Referral Hospital from February 1 to March 30, 2018. The University of Gondar Referral Hospital is found in the North Gondar administrative zone, Amhara National Regional State, 740 km far from Northwest of Addis Ababa (the capital city of Ethiopia). Based on projections of the latest population and housing census report, the total population size of Gondar town was estimated to be 324,000. Currently, Gondar town has one referral hospital and five government healthcare centers. The University of Gondar Referral Hospital is a teaching hospital that serves more than five million people in the central Gondar zone and peoples of the neighboring zones.

### 2.2. Population

#### 2.2.1. Source Population

All diabetes patients who visited the University of Gondar Referral Hospital chronic illness clinic during the study period are the source population.

#### 2.2.2. Study Population

All patients with diabetes who visited the University of Gondar Referral Hospital chronic illness clinic during the study period that fulfilled the eligibility criteria are the study population.

#### 2.2.3. Eligibility Criteria

Diabetes patients who were willing and able to provide written informed consent were included in the current study, whereas history or presence of clinically significant chronic disease other than diabetes mellitus, positive hepatitis B (hepatitis B surface antigen) and/or hepatitis C (hepatitis C antibody) serology, positive HIV serology, and other conditions (including drug abuse, alcohol abuse, or psychiatric patients) was excluded from the current study.

#### 2.2.4. Sample Size and Sampling Technique

Single population proportion formula was utilized by taking the proportion of intestinal parasites among diabetes patients. By considering 18.7% prevalence of intestinal parasites among diabetes patients in a study done in Benin City, Nigeria [10], using a 95% level of confidence and 5% margin of error, the sample size was

(1) $$\begin{array}{c} n=\frac{{\left(z\alpha /2\right)}^{2}*P*\left(1-p\right)}{w^{2}}=\frac{{\left(1.96\right)}^{2}*\left(0.187\right)\left(0.813\right)}{{\left(0.05\right)}^{2}}=234, \end{array}$$

where *P* is the best estimate of a population proportion, *z*_*a*/2_ is the value under the standard normal table for the given value of confidence level, and *α* is the level of significance which can be obtained as 1-confidence level.

A systematic random sampling technique was used to select the total sample size of 234 diabetes mellitus patients.

### 2.3. Data Collection and Laboratory Methods

#### 2.3.1. Sociodemographic Data

First, patients were asked to give informed consent. Then, sociodemographic characteristics and clinical data were collected by group members of the researcher using a semistructured questionnaire. The questionnaire was prepared in English and translated to Amharic for study participants who arrived at the time of sample collection.

### 2.4. Sample Collection and Process

A 5 gram of fresh stool specimen was collected from each of the patients with a suitable container which is clean, dry, and leak-proof, clearly labeled with the time and date of collection and name of the study participant. Then, the stool sample was subjected to a direct saline wet mount method and formal ether concentration technique for the identification and detection of intestinal parasites.

### 2.5. Data Management and Quality Control

The questionnaire was pretested in an area different from the study area for its accuracy and consistency before actual data collection. Appropriate training was given for data collectors about the objective and relevance of the study, confidentiality issues, study participant's rights, consenting, techniques of interview, and laboratory test procedures and their quality control. Sociodemographic and clinical data were collected by a member of the researcher under the supervision of investigators/advisors.

The quality of the test result was maintained strictly by following laboratory standard operating procedures and laboratory manuals of the University of Gondar Hospital Laboratory starting from the preanalytic phase of sample collection up to the postanalytical phase of result interpretation. The collected sample was subjected to analysis in the laboratory immediately after collection or preserved in the refrigerator. Any laboratory tests were analyzed after quality control is run, and the method is ensured to be safe. Furthermore, the investigators closely followed and frequently checked the data collection process to ensure the completeness and consistency of the collected data.

### 2.6. Data Analysis and Interpretation

Data were checked manually for its completeness and clarity and edited for its consistency. After cleaning and coding, data was entered and analyzed by SPSS version 16 statistical package. Descriptive statistics of frequency distributions, summary, and variability measurements were used. Logistic regression models were used to determine the association between dependent and independent variables. *p* value < 0.05 was considered as statistically significant.

### 2.7. Ethical Consideration

Ethical clearance was obtained from the University of Gondar College of Medicine and Health Sciences, School of Biomedical and Laboratory Sciences ethical clearance committee. Permission to conduct the study was also obtained from the University of Gondar Referral Hospital. Additionally, after explaining the importance, purpose, and procedure of the study briefly, written consent was obtained from study participants. Anyone not willing to take part in the study had full right to withdraw, and the confidentiality of the study participants was also maintained by using codes instead of personal identifiers. Any study participants who were positive for intestinal parasite were referred to the concerned physicians for treatment and better management.

## 3. Result

### 3.1. Background Characteristics of Study Participants

A total of 234 diabetes mellitus patients were enrolled in this study, of these 163 were males. Ninety (38.46%) were in the age range of 31-45 years. The mean age of the participants was 39.15. The majority, 217 (92.74%), of study subjects were Christians; 166 (70.94 4%) were married; and 129 (55.13%) were urban residents (Table 1).

### 3.2. Prevalence of Intestinal Parasites among Diabetes Mellitus Patients

**Figure 1:**
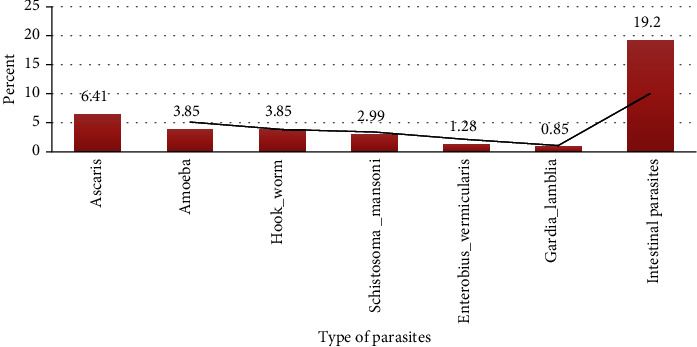
Type and frequency of intestinal parasites among diabetes mellitus patients at the University of Gondar Hospital, Gondar, Ethiopia, 2018.

Forty-five (19.2%) of the study participants had an intestinal parasitic infection. Among these, 15 (6.41%) of the study participants were found to be infected with *Ascaris lumbricoides* followed by *Entamoeba histolytica*/*dispar* 9 (3.85%) and *Hookworm* 9 (3.85%) (Figure 1).

### 3.3. Factors Associated with the Prevalence of Intestinal Parasites

It was found that poor educational background, poor hygiene and sanitation, and inappropriate latrine usage were found to be significantly associated with intestinal parasitic infections among diabetic patients. Though residence was found to be significant in the bivariate analysis, it was found to be insignificant in the multivariate analysis. Those diabetic patients unable to read and write had 3.62 times (AOR = 3.62; 95% CI (1.038, 12.65)) higher odds of being infected with intestinal parasitic infection than the literate category (above high school). Diabetes patients having inappropriate latrine usage had 5.41 times (AOR = 5.41; 95% CI (1.43, 20.56)) higher odds of parasitic infection than those who use latrine properly. Besides, diabetic patients having poor sanitation and hygiene had 4.67 times (AOR = 4.67; 95% CI (1.82, 12.07)) higher odds of parasitic infection than the counterparts (Table 2).

## 4. Discussion

Intestinal parasites are among the most public health important pathogens that can cause infections in immune-compromised individuals. These organisms are capable of infecting individuals with impaired cellular immunity. Emerging intestinal parasites have gained increasing attention as important opportunistic pathogens responsible for clinically important infections in immune-compromised patients. Diabetes mellitus patients have been reported to be immune-compromised so that the clearance of intestinal parasites might have been impaired in diabetes mellitus patients [11].

Forty-five (19.2%) of the study participants in the current study were infected with the intestinal parasitic infection which is in line with the prevalence reported in the southwestern part of Nigeria (18.7%) [10]. But higher than the prevalence reported in Cameroon (10%) [12], Tehran province in Iran has 15.6% [13]. However, the result of this study was lower as compared to the study conducted in Turkey (31.8%) [14]. The difference in geographical location, the diagnostic technique used, the number of study participants included in the study, and the study population might be the possible reason for the variation in the prevalence of intestinal parasites.

In the current study, the prevalence of parasitic infections was twice higher in diabetes mellitus patients from rural areas 30 (66.7%) than from the urban areas 15 (33.3%). This might be due to lack of awareness, absence of latrine pit, or improper use of latrine in rural areas. Besides, individuals from rural areas are more likely to be engaged in farming and domestic work which exposes them to these intestinal parasite infections. However, this result differed from the findings reported in Arba Minch, Ethiopia, where the prevalence of parasitic infections was higher from the urban areas 33 (15.3%) than in rural areas 9 (4.2%) [15]. This might be due to the difference in hygiene and sanitation usage in these two particular areas and could be due to the migration of DM patients from rural to urban areas so that overpopulation can result in a high prevalence of intestinal parasites [11, 16].

In this study, a total of six different intestinal parasites were identified from which two of them were intestinal protozoans (*Entamoeba histolytica*/*dispar* 9 (3.85%) and *Giardia lamblia* 2 (0.9%)) and the remaining four were helminths (*Ascaris lumbricoides* 15 (6.41%), *Hookworm* 9 (3.85%), *Schistosoma mansoni* 7 (3%), and *Enterobius vermicularis* 3 (1.3%)). *Ascaris lumbricoides*, *Entamoeba histolytica*, and Hookworm were the most prevalent parasites in the current study. This finding differs from the study carried out in Egypt with three different intestinal parasites (*Entamoeba histolytica*/*dispar* 13 (39.4%), *Ascaris lumbricoides* 1 (3%), and no Hookworm infection) were identified [17].

The present study showed that level of education, hygiene, sanitation, and latrine usage were significantly associated with the prevalence of intestinal parasitic infections in diabetes mellitus patients. Diabetic patients who were unable to read and write were 3.62 times (AOR = 3.62; 95% CI (1.038, 12.65); *p* = 0.043) more likely to be infected with intestinal parasitic infection than the literate category (above high school). In contrast to this finding, a study conducted in Iran showed that education (AOR = 2.87; 95% CI (0.66, 12.38); *p* = 0.157) was not significantly associated with the prevalence of intestinal parasitic infections among diabetes mellitus patients [9]. This might be due to the difference in the level of awareness of intestinal parasite transmission in the population.

This study showed that diabetes patients having poor sanitation and hygiene had 4.67 times (AOR = 4.67; 95% CI (1.82, 12.07); *p* = 0.001) higher odds of parasitic infection than their counterparts. This might be due to elevated intestinal parasitic infections that have been recorded in developing countries because of the low literacy rate; the paucity of potable water resulted in poor hygiene and sanitation [18, 19].

The current study showed that diabetes patients having inappropriate latrine usage had 5.41 times (AOR = 5.41; 95% CI (1.43, 20.56); *p* = 0.013) higher odds of parasitic infection than those who use latrine properly. This may be probably due to open defecation that resulted in feco-oral contamination. This is in line with the study conducted in the southwestern part of Nigeria where poor latrine usage (*p* = 0.0001) was significantly associated with the prevalence of intestinal parasites among diabetes mellitus patients [10, 11]. The most prevalent parasites isolated from stool samples were *Ascaris lumbricoides*, *Entamoeba histolytica*, and *Hookworm* infection. The results of our study showed that there was an association of intestinal parasitic infections in diabetic patients. The presence of intestinal parasites may cause a hazardous effect on the health of diabetic patients, and therefore, its risk should be considered. As a limitation, the current study used only a wet mount and formal-ether concentration technique for microscopic diagnosis of intestinal protozoa and helminths. Additional laboratory techniques such as Modified Ziehl-Neelsen Acid-fast stain and molecular methods were not used in the current study.

In conclusion, the overall prevalence of intestinal parasitic infection among diabetes mellitus patients was relatively higher than the previously reported studies. *Ascaris lumbricoides*, *Entamoeba histolytica*, and Hookworm infection were highly prevalent intestinal parasites recovered. Poor educational background, poor hygiene and sanitation, and inappropriate latrine usage were significantly associated with the prevalence of intestinal parasitic infections among diabetes mellitus patients. Therefore, diabetic patients should be screened routinely for intestinal parasites and treated for their overall wellbeing. Besides, health education should be provided for diabetes mellitus patients to improve awareness about parasite infection for preventive purposes.

## Figures and Tables

**Table 1:** Sociodemographic characteristics of diabetes mellitus patients at the University of Gondar Hospital, Gondar, Ethiopia, 2018.

Variable		Frequency	Percent
Sex	Male	163	69.66
	Female	71	30.34

Age	15-30	70	29.9
	31-45	90	38.46
	>45	74	31.62

Religion	Christian	217	92.74
	Muslim	17	7.26

Marriage	Single	44	18.8
	Married	166	70.94
	Divorced	14	5.98
	Separated	10	4.27

Residence	Urban	129	55.13
	Rural	105	44.87

Education	Unable to read and write	76	32.48
	Primary school	50	21.37
	High school	34	14.53
	Above high school	74	31.62

Occupation	Government employed	55	23.5
	Private employed	47	20.09
	Daily laborer	17	7.26
	Farmer	66	28.21
	Merchant	38	16.24
	Student	11	4.7

Monthly income	<500	23	9.83
	500-1500	87	37.18
	1501-3000	46	19.66
	3001-5000	52	22.22
	>5000	26	11.11

**Table 2:** Factors associated with intestinal parasitic infection among diabetic patients at the University of Gondar Hospital, Gondar, Ethiopia, 2018.

Variables		Intestinal parasite (%)		COR 95% CI	AOR 95% CI	*p* value
		Present	Absent			
Sex	Male	32 (13.7)	131 (55.9)	1.09 (0.53, 2.23)		
	Female	13 (5.56)	58 (24.8)	1		

Age	15-30	13 (5.56)	57 (24.4)	1		
	31-45	19 (8.1)	71 (30.34)	1.07 (0.46, 2.50)		
	>45	13 (5.56)	61 (26)	1.26 (0.57, 2.75)		

Residence	Urban	15 (6.4)	114 (48.7)	1	1	
	Rural	30 (12.8)	75 (32)	3.04 (1.53, 6.30)	0.27 (0.62, 1.20)	0.087

Education	Unable to read and write	26 (11.1)	50 (21.36)	5.89 (2.26, 15.39)	3.62 (1.038, 12.65)	0.043
	Primary school	7 (2.9)	43 (18.4)	1.85 (0.58, 5.86)	1.22 (0.31, 4.85)	0.774
	High school	6 (2.56)	28 (11.9)	2.43 (0.72, 8.18)	1.20 (0.55, 7.27)	0.293
	Above high school	6 (2.6)	68 (29.1)	1	1	

Hygiene and sanitation	Sometimes	38 (16.2)	86 (36.7)	6.50 (2.76, 15.30)	4.67 (1.82, 12.07)	0.001
	Always	7 (2.9)	103 (44)	1	1	

Latrine	Yes	16 (6.84)	141 (60.3)	1	1	
	No	29 (12.4)	48 (20.5)	5.32 (2.63, 10.64)	5.41 (1.43, 20.56)	0.013

Type of diabetes	Type one	36 (15.4)	149 (63.7)	1.07 (0.48, 2.41)		
	Type two	9 (3.85)	40 (17.1)	1		

Duration of diabetes (in year)	<5 year	20 (8.5)	95 (40.6)	0.56 (0.23, 1.39)		
	5-10 year	16 (6.84)	70 (29.9)	0.61 (0.24, 1.56)		
	>10 year	9 (3.85)	24 (10.26)	1		

Type of antidiabetic drug	Injectable	37 (15.8)	150 (64.1)	1.20 (0.52, 2.79)		
	Oral	8 (3.42)	39 (16.7)	1		

COR: crude odds ratio; AOR: adjusted odds ratio.

## Data Availability

Data is available upon request from the corresponding author.
